# Minimal Effects of an Invasive Flowering Shrub on the Pollinator Community of Native Forbs

**DOI:** 10.1371/journal.pone.0109088

**Published:** 2014-10-24

**Authors:** Y. Anny Chung, Laura A. Burkle, Tiffany M. Knight

**Affiliations:** 1 Department of Biology, Washington University in St. Louis, St. Louis, Missouri, United States of America; 2 Department of Biology, Albuquerque, New Mexico, United States of America; 3 Department of Ecology, Montana State University, Bozeman, Montana, United States of America; University of New South Wales, Australia

## Abstract

Biological invasions can strongly influence species interactions such as pollination. Most of the documented effects of exotic plant species on plant-pollinator interactions have been observational studies using single pairs of native and exotic plants, and have focused on dominant exotic plant species. We know little about how exotic plants alter interactions in entire communities of plants and pollinators, especially at low to medium invader densities. In this study, we began to address these gaps by experimentally removing the flowers of a showy invasive shrub, *Rosa multiflora*, and evaluating its effects on the frequency, richness, and composition of bee visitors to co-flowering native plants. We found that while *R. multiflora* increased plot-level richness of bee visitors to co-flowering native plant species at some sites, its presence had no significant effects on bee visitation rate, visitor richness, bee community composition, or abundance overall. In addition, we found that compared to co-flowering natives, *R. multiflora* was a generalist plant that primarily received visits from generalist bee species shared with native plant species. Our results suggest that exotic plants such as *R. multiflora* may facilitate native plant pollination in a community context by attracting a more diverse assemblage of pollinators, but have limited and idiosyncratic effects on the resident plant-pollinator network in general.

## Introduction

The introduction of exotic plant species with a showy floral display has the potential to alter plant-pollinator interactions of the resident community. A recent meta-analysis reported that overall, exotic plant species tend to negatively affect the pollination and reproductive success of native plant species [1] Specifically, the presence of exotic plant species can decrease the diversity and abundance of pollinators that occur in the area [2], decrease the pollinator visitation rates observed on native plants [3]–[5], and increase interspecific pollen transfer for native plants [6]. However, in some cases exotic plants positively influence the pollination of native plant species by attracting a greater abundance and diversity of pollinators to the area [7] and increase pollinator visitation rate to some native plant species [8].

It is possible that the greater support in the literature for negative effects of exotic species on native plant pollination is because researchers have largely focused on communities containing moderate to high densities of exotic plants, where one would expect to see strong effects of exotic plant presence. The majority of exotic species, however, do not achieve high densities [9], [10]. Therefore, investigations of exotic species invasions in habitats where they are not dominant provide additional insight into the spectrum of possible exotic plant effects on native plant-pollinator communities.

Interactions between plants and pollinators are typically generalized within an assemblage and across multiple taxonomic ranks [11], and thus considering the entire network of interactions in the community can provide mechanistic information about how exotic plants alter the visitation rates and visitor diversity for resident plant species. A few such studies exist [2], [12]–[14], and these find that the effects of exotic plants on native plant pollination are variable across focal exotic species and across native plants species within a network. For example, exotic *Carpobrotus* (Aizoaceae) species had overall facilitative effects on native plants by increasing the diversity and visitation frequency of pollinators, whereas exotic *Opuntia* (Cactaceae) species resulted in overall lower pollination success of natives [15]. Moreover, the effects of exotic plants on native plant pollination are not consistent across species, with positive, negative and neutral effects observed across species within a network [16].

In cases for which exotic plants decrease the visitation of native plant species, competition for floral resources is typically invoked as the causal mechanism. However, few studies experimentally remove flowers to test whether the presence of exotic flowers, as opposed to other mechanisms (e.g., competition with exotic plants for other resources that might affect floral display), alter visitation in native plant species. To our knowledge, only one study [7] has taken an experimental approach to assessing the community-level effects of a dominant exotic plant species on native plant-pollinator interactions. Other studies either compared invaded and uninvaded sites [15], [16] or sites along a natural invasion gradient [14]. Observational studies utilizing sites that naturally vary in invasion intensity may be confounded by underlying abiotic gradients (e.g. soil texture, nutrient levels, pH, and disturbance [17], [18]) and biotic gradients (e.g. herbivory, competition; reviewed in [19]) that may influence the quality and quantity of floral traits and rewards [20], [21].

Plant-pollinator networks are asymmetric in the distribution of interactions between species: generalists tend to interact with both generalists and specialists, while specialists mostly interact with generalists [22]. Previous research suggests that exotic plant species are typically generalized in their pollination and are readily incorporated into plant-pollination networks by native generalist pollinators [12], [23]. However, because these native generalist pollinators are important visitors to specialized native plant species, exotic plants are expected to have the strongest effects on specialized native plant species [24], [25]. These effects could be either positive (e.g., if exotic plants attract more pollinators to the area) or negative (e.g., if exotic plants compete with native plants for resident pollinators) in direction.

In this study, we experimentally investigated the community-level effects of an exotic flowering shrub, *Rosa multiflora*, on plant-bee interactions in habitats where it was present but not dominant. Specifically, we asked: (1) Does *R. multiflora* attract more diverse and frequent visits from the local bee pollinator species pool compared to co-flowering natives? (2) To what degree does *R. multiflora* alter bee species visitation rate, richness, and community composition to the native plant community? (3) Is *R. multiflora* visited by more generalist bee species compared to the native plant species in the community?

## Methods

### Study System

This study was conducted in Carlinville, Illinois, USA. We chose this study area because it has a long history of plant-pollinator research and the pollinator fauna is very well-described [26]. We initially conducted a pilot study of exotic plant species in old fields, woodland edges, and prairie remnants in order to identify an exotic plant species that attracts a diversity of pollinators and thus has the potential to influence the pollination ecology of co-flowering native plants (Table S1 in Methods S1).

### Focal exotic species: *Rosa multiflora*

In the pilot study, we studied the diversity and visitation rate of pollinators to flowers of nine exotic plant species. Of these, *Rosa multiflora* received the highest diversity of pollinators, as well as a moderate visitation rate (Figure S1 in Methods S1). Hence, we selected *R. multiflora* as the focal exotic species for further study. *Rosa multiflora* is an exotic invasive subshrub (a low shrub with partly herbaceous stems) native to East Asia that has spread throughout the United States [27]. It is listed as a noxious weed in 12 states in the USA [27], and is a common invader of open woodlands, often forming dense undergrowth that excludes other plant species [28]. *Rosa multiflora* was introduced to Illinois in the 1940s as plantings for farm hedge [29] and has since invaded the woodlands surrounding Carlinville. It produces abundant flowers, up to 200 flowers per panicle and multiple panicles per plant [29], as well as pollen and scent to attract pollinators. Furthermore, *R. multiflora* shares pollinators with co-flowering native species [30], making it an ideal species for the purposes of our study.

### Experimental removal of *R. multiflora* flowers

Five study sites in Carlinville, IL were chosen in the spring of 2010 in wooded areas with naturalized populations of the focal exotic plant, *R. multiflora*. Two sites were in the Culp Conservancy Woods, which is privately owned and registered with the Illinois Nature Preserves Commission, from which research permits were obtained. The remaining research sites were on private land owned by the Swiatkowski family in Carlinville, IL, who should be contacted for future permissions. No endangered or protected species were involved in this study.

All sites had similar native understory flora including the herbaceous dominant species *Sanicula odorata* (Apiaceae), and lower densities of *Monarda bradburiana* (Lamiaceae), and *Blephilia ciliata* (Lamiaceae) (Table 1). *Rosa multiflora* comprised 3–43% (0.88–8.9 flowers/m^2^) of the total floral density at peak bloom across sites. The distance between sites was 300 m to >10 km. As the mean foraging distance for highly mobile and widely-foraging bumble bees are c. 275 m [31], these sites were spaced far enough apart to minimize individual bee movement among sites. Each site was split into two paired treatment plots of 50 m×20 m, separated by a 50 m buffer zone. Since bees can fly between plots, this spatial scale of study allows bee foraging choice to potentially influence visitation to plant species in each treatment. Prior to treatment manipulation, the two plots and buffer zone were all visually similar in floristic composition. In the control treatment, blooming *R. multiflora* individuals were left unmanipulated, whereas in the removal treatment and buffer zone, all flowers and buds of *R. multiflora* individuals were clipped. Only the floral parts were removed to control for potential shade, moisture, and structural effects of the rose shrubs on other plant species and on pollinator behavior. *Rosa multiflora* individuals in the removal treatment and buffer zone were surveyed every other day throughout the experimental period, and buds were removed as necessary.

**Table 1:** Study plant species and their attributes.

Species	Family	Life history and growth form^1^	Floral morphology	Breeding system	Rounded I-Rank^2^
***Blephilia ciliata***	Lamiaceae	Perennial Forb/herb	Closed	animal-pollinated	NA
***Monarda bradburiana***	Lamiaceae	Perennial Forb/herb	Closed	animal-pollinated	NA
***Sanicula odorata***	Apiaceae	Perennial Forb/herb	Open	mixed	NA
***Rosa multiflora***	Rosaceae	Perennial Vine/subshrub	Open	animal-pollinated	Med

1 USDA PLANTS database (USDA NRCS 2013).

2 U.S. Invasive Species Impact Rank (natureserve.org).

To compare visiting bee species richness and composition to native plants between treatments, we conducted pollinator surveys in each plot throughout the bloom period of *R. multiflora* (May 18–26, 2010) in days without rain and ≤60% cloud cover to ensure observation of pollinator activity (personal observation). On each observation day, one site was surveyed in the morning and a second site in the early afternoon. To account for potential biases due to variation in atmospheric conditions, we rotated the time of day at which each experimental site was surveyed such that sites were equally observed in both morning and afternoon periods throughout the course of the study. At each site, the control and treatment plots were observed simultaneously. Surveyors observed co-flowering native species and *R. multiflora* in each plot and collected all insect visitors to the reproductive parts of all flowering individuals (native and exotic). Observation times for each species were recorded. On each sampling day, we also recorded floral abundances in two 20 m×2 m band transects per plot. Only bee visitors (Hymenoptera, Apoidea), which included 78% of all individuals collected and >50% of all visitors to each focal plant species, were considered in the analyses. Other visitors collected but not considered in the analyses included beetles (Coleoptera, 12%), flies (Diptera, 8%), wasps (Hymenoptera, Apocrita, 1%), and other insects (1%). We chose to focus only on bee visitors because they are known to be the most effective pollinators [32], and we were interested in potential links between exotic plant invasion and native plant reproductive success. Across sites, we spent 24.8 observation hours in *R. multiflora* removal plots, and 41.2 hours in the control plots. More time was necessary in the control plots to gain a representative sample of the bees visiting *R. multiflora* as well as the native plant species. Total observation time spent on native plant species was similar among treatments (*t* = −0.45, *df* = 4, *p* = 0.68).

### Statistical Analyses

To ascertain whether floral density was similar among treatments, we compared native and total (native and *R. multiflora* combined) floral densities between treatments. Floral density data were natural log-transformed to meet normality assumptions.

We asked whether *R. multiflora* attracted more frequent visits from the local bee pollinator species pool compared to co-flowering natives. Visitation rate for each plant species was calculated as the number of bees caught in each plot per hour per flower. A visitation rate was calculated for each observation day at each site where that plant was present. Due to unequal sample sizes in calculated visitation rates among plant species, we performed a randomization test in lieu of an ANOVA to compare bee visitation rates among focal plant species. Instead of testing whether there is a difference in visitation among plant species as in an ANOVA, this randomization test investigates each pairwise comparison between species. In the randomization test, plant species identity was randomly shuffled across all observations for all plants to assign visitation rates as previously calculated above. The difference in mean visitation rate to each plant species was then calculated for all pairwise comparisons between plant species in each run. This was repeated 10000 times for a null estimate of the expected and 95% confidence interval of difference in visitation rate to each plant species pair. Observed differences were then compared to the expected to determine whether it was significantly larger than expected (outside 95% CI), indicating that the plant species in that pairwise comparison had significantly different visitation rates from each other.

We asked whether *R. multiflora* attracted more diverse bee visitors compared to co-flowering natives. Richness of visitors to each plant species was rarefied to control for differences in the number of bee individuals sampled across plant species (ECOSIM [33]). Using all data across treatments, bee visitor richness between plants was compared using 95% CI calculated from rarefaction variance estimates.

To determine whether the presence of *R. multiflora* altered the richness and frequency of bee visitation to co-flowering natives, we compared visitation rates and rarefied richness of bee visitors to only native plants between treatments using paired t-tests. Bee visitation data (species identity and number of individuals caught) were pooled across all native plant species in each plot and paired by experimental site. We then calculated visitation rates to all native plants in each plot using the same methods as above. Tests on each native plant species individually were not possible because not all plant species were present at all sites in all plots. Bee visitor richness was rarefied to control for variation in different numbers of individuals sampled in each plot (ECOSIM [33]). We calculated 95% confidence intervals and standard deviations around rarefied estimates. To further investigate if differences in rarefied bee visitor richness between control and treatment plots could be explained by *R. multiflora* invasion intensity at each site, we conducted a simple linear regression using the fraction of *R. multiflora* flowers of total floral density at each site during peak bloom as a measure of invasion intensity.

To compare the composition of bee visitors to all native plants between control and treatment plots, we used nonmetric multi-dimensional scaling (NMDS) with Bray-Curtis dissimilarity coefficients [34] to characterize the composition of bee visitors. We then conducted a permutational multivariate analysis of variance using Bray Curtis dissimilarity to compare plot-level bee visitor composition to native plant species among treatments and sites. In addition, we compared group dispersions among treatments to determine effects of *R. multiflora* flower removal on dispersion (variance) in bee community composition.

To test whether generalist bee species were more likely to visit *R. multiflora* than specialist bee species, we investigated if bee diet breadth was associated with the likelihood of its visiting *R. multiflora*. Pollinator specialization (monolectic, oligolectic, and polylectic) is traditionally defined as the taxonomic diversity (number of genera/family used as plant hosts) [35], as floral morphologies and rewards are known to be phylogenetically conserved [11], [36], [37]. Therefore, we estimated bee diet breadths using the number of plant families each bee species is known to visit [38]. Nineteen bee individuals totaling 12 species were observed visiting *R. multiflora*. We compared the weighted mean diet breadth of visitors to *R. multiflora* to that expected by chance by randomly choosing 19 of the total 272 individuals observed in the experiment without replacement, and calculating their mean diet breadth. This sampling scheme was replicated 1000 times so that 95% confidence intervals could be calculated. All analyses were performed in R (R Development Core Team. Version 3.0.0) unless otherwise noted.

## Results

**Figure 1:**
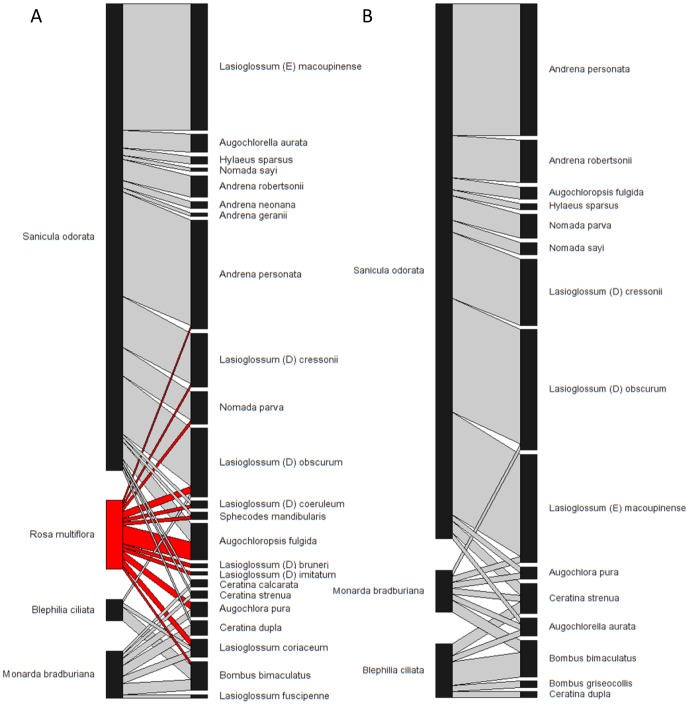
Plant-bee interaction networks of all study sites. Plant-bee networks of all sites in Carlinville, Illinois, U.S.A. in **A**) control and **B**) removal plots. Bee species are represented by the boxes on the right and plant species on the left. Grey bars connecting boxes depict observed interactions between bees and plants. Interactions with the focal exotic *Rosa multiflora* are highlighted in red. Box and bar widths are proportional to the number of recorded interactions.

We captured and identified 272 individuals of bee visitors, totaling 24 species, from control and removal treatments combined (Fig. 1, see also Data S1). Total floral density was marginally higher (average increase of 2.71 flowers/m^2^) in the control plots with *Rosa multiflora* present compared to the removal plots (*t* = 2.13, *df* = 10, *p* = 0.06). The floral density of native plants was not significantly different between treatments (*t* = 0.57, *df* = 10, *p* = 0.58). This suggests that the difference in total floral density between treatments was driven by the presence of *R. multiflora*, confirming the efficacy of our treatment, and that any differences in bee visitation between treatments could be attributed to *R. multiflora* floral density.

**Figure 2:**
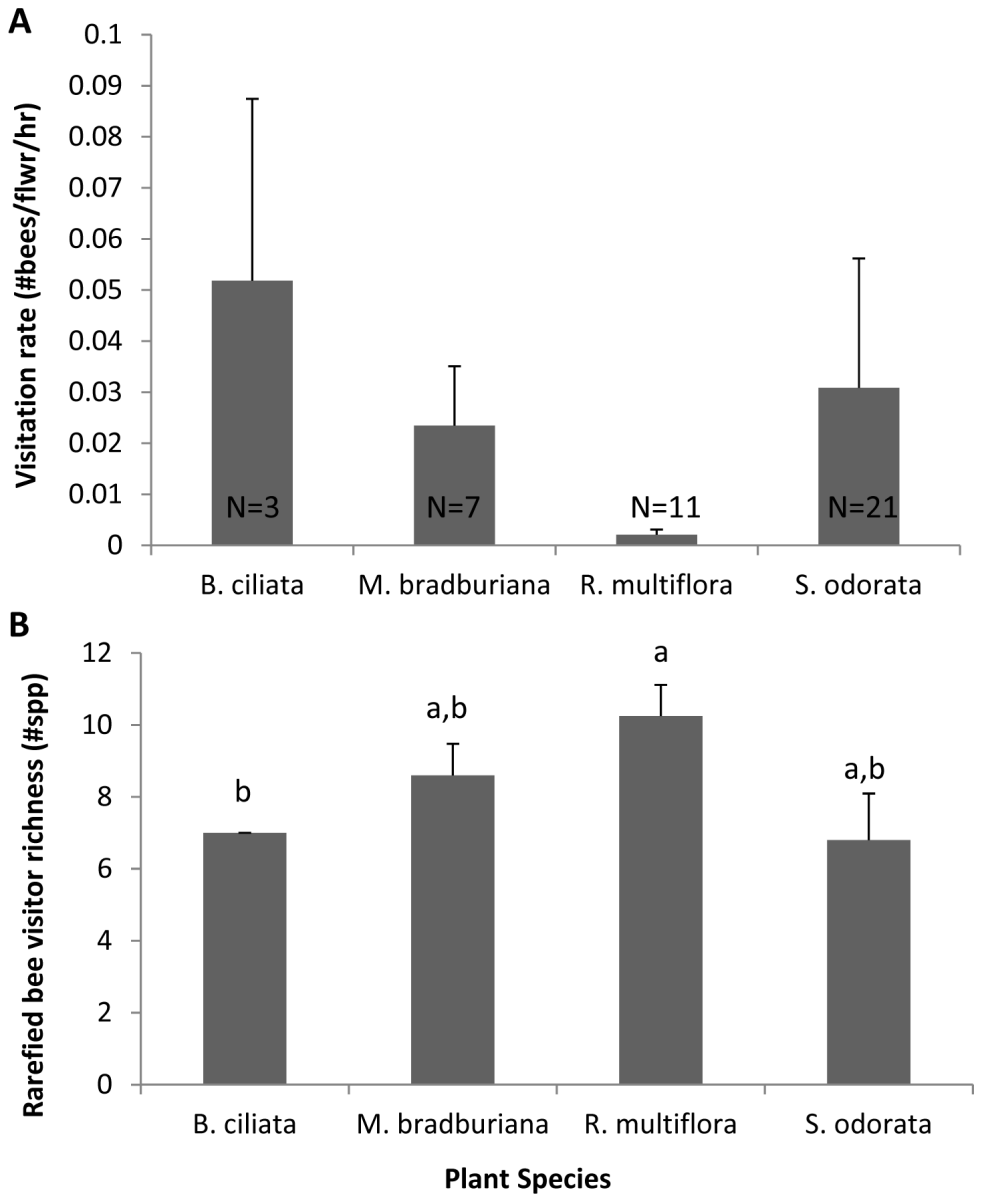
Bee visitation rate and visitor richness to *R. multiflora* and native forbs. **A**) Visitation rate of bee visitors to *R. multiflora* and native plants across all sites. Errors indicate SE. Native plants in control and treatment plots were combined. All estimates of visitation rate significantly differed from each other (p<0.05) based on a randomization test. Sample sizes for each estimate are labeled at the bottom of each bar. **B**) Rarefied bee visitor richness to each plant species across all sites. Letters indicate groupings by 95% confidence intervals.

The mean visitation rate of bee visitors to *R. multiflora* across sites was less than 10% than that of all other native plant species (*p*<0.05, Fig. 2A), but the diversity of visitors to *R. multiflora* was >19% higher relative to native plant species (Fig. 2B). *Rosa multiflora* in control plots were visited by 12 bee species, six of which were species in the genus *Lasioglossum*. Of the 12 bee species found visiting *R. multiflora*, two species (*Lasioglossum bruneri* and *Lasioglossum imitatum*) were only observed to visit *R. multiflora* and no native plant species. It is possible that bee species might also appear relatively specialized because they are rare within the community. *Lasioglossum bruneri* was observed only once (visiting *R. multiflora*) throughout this study, but museum records indicate that it is known to visit host plants across 13 plant families [38]. so it is unlikely a specialist on *R. multiflora*. From the historic records of the area, *L. imitatum* has been known to visit native plant species in the community such as *Blephilia ciliata* [26], but it was only observed once in our study. The remaining 10 bee species that visited *R. multiflora* were shared with at least one other native plant.

**Figure 3:**
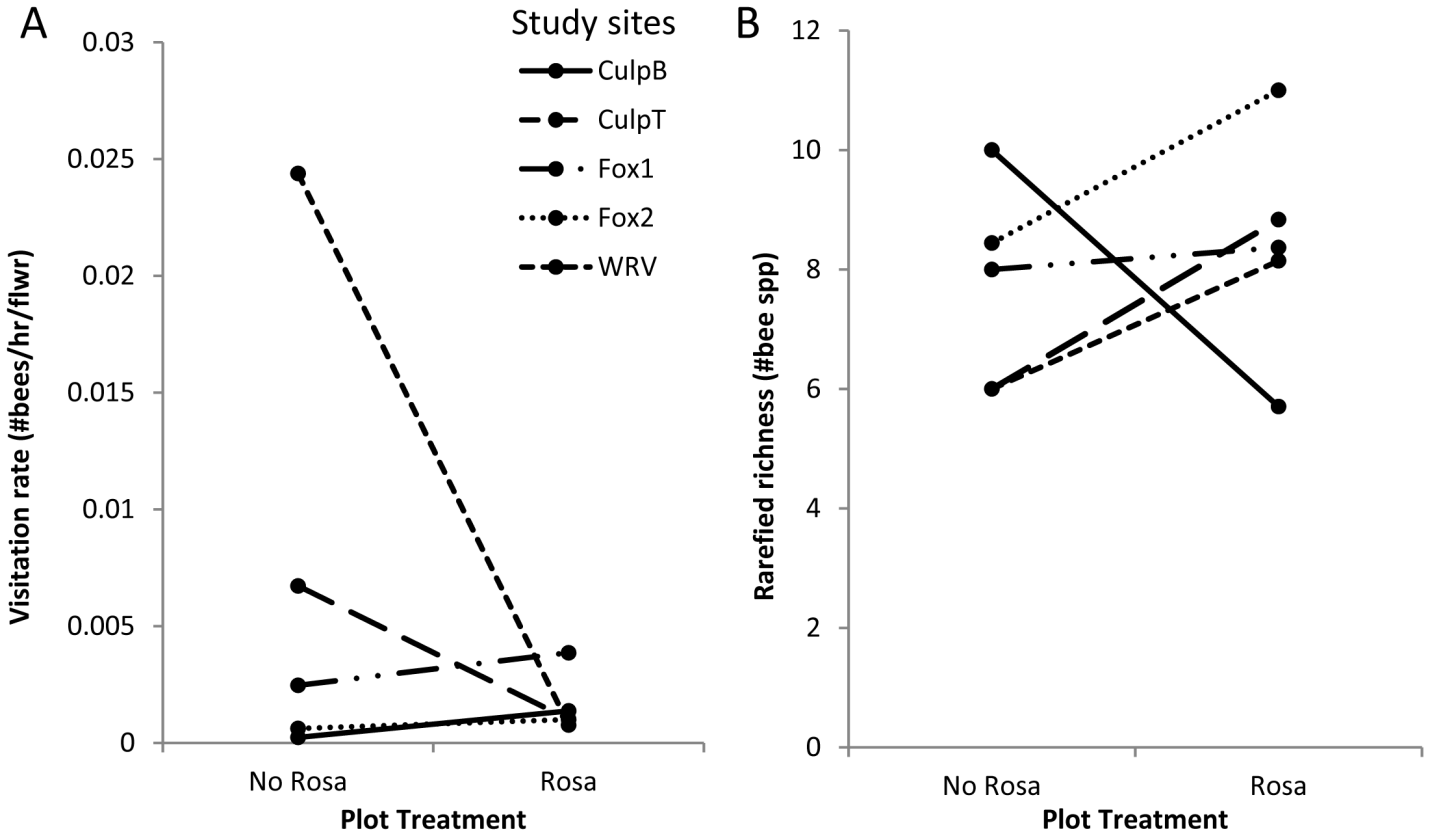
Comparison of bee visitation rate and visitor richness in control and treatment plots. Visualization of paired t-tests in **A**) bee visitation rate and **B**) bee visitor richness to native plants in paired plots at each site. The bee visitor richness trend at CulpB runs contrary to the other four sites.

**Figure 4:**
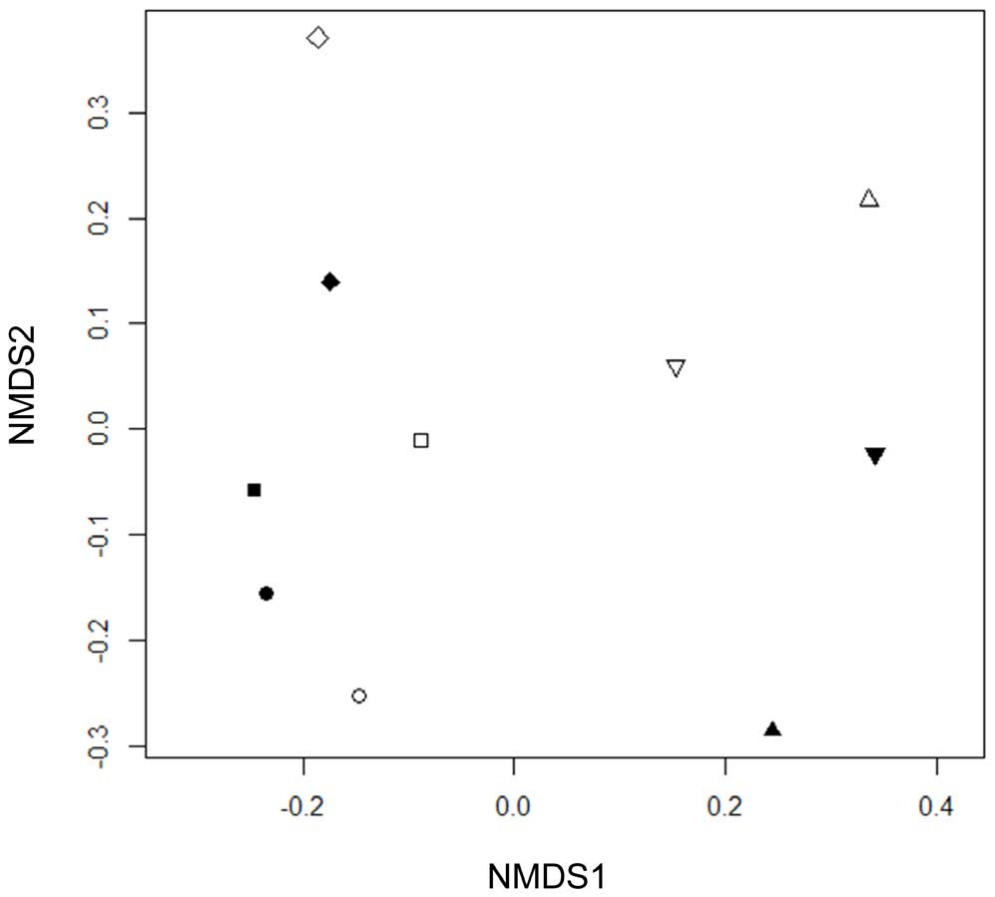
Comparison of species community composition of bee visitors to native forbs across plots and sites. NMDS of all control and treatment plots. Control plots (*R. multiflora* flowers present) are filled, and treatment plots *(R. multiflora* flowers absent) are open; sites are represented by different symbols. Pollinator community composition was better predicted by experimental site, and the *R. multiflora* removal treatment did not systematically affect pollinator community composition in each plot.

The presence of *R. multiflora* flowers had no effect on bee visitation rates to native plant assemblages (*t* = −1.11, *df* = 4, *p* = 0.33; Fig. 3A). However, *R. multiflora* increased rarefied bee visitor richness to the native plant community in four out of the five sites investigated, with an average increase of 12.7% across all sites (Fig. 3B). The overall pattern, however, was not statistically significant in the paired t-test (*t* = 0.55, *df* = 4, *p* = 0.61). The higher bee richness in invaded plots was not explained by *R. multiflora* invasion intensity (proportion of *R. multiflora* flowers of total floral density) at each site (*F* = 0.0003, *df* = 3, *p* = 0.99). Plot-level bee community composition differed significantly among sites (*F* = 2.72, *df* = 4, *p* = 0.01; Fig. 4). However, there were no significant differences in bee community composition (*F* = 1.43, *df* = 1, *p* = 0.21; Fig. 4) and variance in composition (*F* = 0.00004, *df* = 1, *p* = 0.98) between *R. multiflora* treatments.

Bee visitors to *R. multiflora* had a significantly wider mean diet breadth (MDB) than expected by random chance (expected MDB and 95% CI: 23.78 (19.16–28.79); observed MDB: 31.47).

## Discussion

Our results showed that 1) *Rosa multiflora* was visited by the richest assemblage of bee species of all plants studied, but at the lowest visitation frequency, 2) the presence of *R. multiflora* did not alter visitation rates or the diversity and composition of visitors to native plant species and 3) *R. multiflora* received visits from bees with wider diet breadths (more generalist) than expected by chance. We discuss the implications of these findings below.

### Bee visitation to *R. multiflora* compared to co-flowering natives

We found the exotic *R. multiflora* to be visited by a diversity of bees but at a low visitation frequency when compared to co-flowering native species. This finding contrasts with others that find higher visitation rates to exotic plant species compared with co-flowering natives [3], [39], [40]. However, such studies often investigate high-density invasions with charismatic floral displays, whereas pollinator visitation has been shown to decline with declining floral density [41], [42]. The high diversity of bee visitors to *R. multiflora* is consistent with other work that shows long-residency exotic plant species accumulate pollinator diversity through time to that equal to native plant species [43], and suggests that *R. multiflora* is fully integrated into the resident plant-pollinator network.

### Effects of *R. multiflora* on resident plant-pollinator interactions

Some of our results suggest that *R. multiflora* presence might facilitate pollination of natives, but in general, we found no significant effect on the visitation of bee visitors to native plants. Specifically, in four of our five study sites we found increased bee visitor richness in the presence of *R. multiflora*. Further, in three of our five study sites, we found instances of bee visitors shared by *R. multiflora* and co-flowering natives which occurred only in the control plots and not the treatment plots where *R. multiflora* flowers were removed. It is possible that these species entered the plot because of the presence of *R. multiflora* and also visited the co-flowering native plant species, thus increasing the overall bee visitor richness to native plant species at some sites. The significant differences in bee visitor composition between sites, but not *R. multiflora* removal treatments, likely reflect variation in nesting habitats and the local species pool available at each site. These results also suggest that geographical variation is likely more important than the presence or absence of an exotic plant species in determining bee visitor composition to native floral communities in the early stage of the invasion process. In conclusion, our observations show that the effects of *R. multiflora* presence on the richness of bee visitors were subtle and non-significant.

Other studies that focused on more dominant exotic plant species found more dramatic positive or negative effects of exotic plant presence on the pollination of co-flowering natives (e.g. [7]). The discrepancy between these studies and our research may be because our focal exotic plant species did not dominate the community. Indeed, Kaiser-Bunbury and colleagues [14] found that the influence of exotic flowers on the distribution of interactions in plant-pollinator networks was not seen until more than one third of all flowers in the community belonged to the exotic species. Likewise, an experimental study that manipulated exotic plant density at the neighborhood scale found that at low density, exotic plant presence increased duration of pollinator visits to the focal native, resulting in increased seed set. However, at high densities, exotic plant presence decreased pollinator visitation frequency, duration, and seed set of focal natives [44]. *Rosa multiflora* does not generally occur in high densities in forest habitats such as the ones that were the focus of our study that contain several co-flowering native species. However, in more open habitats, *R. multiflora* can dominate the floral community (personal observation). While it is possible that in such open habitats that *R. multiflora* might have different effects on pollination of native plants compared to the results of this study, we note that open habitats tend to have few co-flowering native plant species. Thus, we think it is possible that *R. multiflora* might have a minimal effect on native species through altering their pollination in all of the habitats it invades.

Exotic plant introductions are likely to have large effects (either positive or negative) on native plant-pollinator networks if many pollinator species are shared and if there is high overlap in flowering phenologies. *Rosa multiflora* shared many bee visitor species with co-flowering native plants at our sites. However, the flowering period of *R. multiflora* (two weeks in mid-late May) was short compared to the flowering period in many native species in these woodland understory habitats. The short flowering period of *R. multiflora* might prevent this species from having strong effects on the pollination and reproductive success of native co-flowering species. Interestingly, many of the other exotic plant species at this study site were shown to have flowering periods were twice as long as those of native plants, and these extended flowering periods have been speculated to explain the high reproductive success of these exotics despite their relatively poor integration into the native pollination network [23].

### *Rosa multiflora* was visited by generalist bee species

*Rosa multiflora* was the most generalist plant species in our study, and was also visited by bees with wider diet breadths (more generalist) than expected by chance from the bee species pool. Previous studies concur that exotic plant species are most likely incorporated into a native plant-pollinator network through generalist pollinators that are less discriminatory in their diet preferences [12], [23], [45]. In this study, we saw that although *R. multiflora* was readily visited by generalist bee species, these visits did not detract from visitation to co-flowering natives. In contrast, the presence of the exotic plant species often facilitated a richer assemblage of bee species visiting native plants. However, theoretical modeling of pollinator foraging strategies in mixed floral stands has shown that as the relative density of a rare flower increases to common, pollinators may switch from a mostly generalist strategy to solely specializing on the most profitable (most common) flower [46]. Therefore, as the relative floral density of *R. multiflora* increases in a habitat, it is possible that its effects on the pollinator richness of co-flowering plants will switch from neutral/facilitative to competitive.

### Implications for exotic plant effects on resident plant-pollinator networks

We are only beginning to understand the implications of species invasions and extinctions for plant-pollinator networks. Plant-pollinator networks are considered robust to perturbations such as random species extinctions due to their nested architecture [47]–[49], and assembly models of plant-pollinator networks suggest that stable communities of interacting species are quickly reached through species additions [50]. In addition, research into the nested architecture of plant-pollinator networks have hinted at the reduced competitive cost of adding new plant species to an established network through interactions with generalist pollinator species [45], [51]. These models are consistent with our findings that *R. multiflora* was visited by bee species with wider diet breadths than expected. Therefore, we hypothesize that when entire communities of plants and pollinators are considered, additions of plant species that are readily visited by existing generalist pollinators to a plant-pollinator network are unlikely to result in plant extinctions due to pollinator-mediated competition, and may even facilitate the inclusion of more pollinator species. In addition, the results of this study and others suggest that relative floral abundance could be an important driver of changes in plant-pollinator interactions (e.g. [52]).

To better understand the impacts of species subtractions (extinctions) and additions (invasions) to plant-pollinator communities, our work highlights the need for more studies that take a manipulative approach to this topic. Such an approach is rigorous, and controls for many of the abiotic factors predisposing a site to invasion. Future work extending an experimental period over several seasons could capture a more complete subset of the existing pollinator community and track the effects of an exotic species on plant-pollinator networks over time. In addition, the wide variation in species richness of bee visitors to *R. multiflora* between sites we observed as well as the variation of invasive plant effects on pollinators reported in the literature suggests that future research should explicitly manipulate invader floral density under natural settings to further understand the range of exotic plant effects on native pollinators and plants.

Our results also point to the prominent role of generalist pollinators in the face of plant species invasions or extinctions. Generalist pollinators readily visit exotic plant species while maintaining original interactions within the native plant-pollinator network. Most importantly, our findings suggest that low density invasion stages of an exotic plant species has limited effects on the resident plant-pollinator network, and may facilitate pollination to co-flowering natives at the community level by attracting a more diverse assemblage of pollinator species.

## Supporting Information

Methods S1 **Exotic plant survey methods and results.**
(DOCX)

Data S1 **Plant-bee interaction and visitation data used in the manuscript.**
(XLSX)
