# Supplementary material for: Minimal Effects of an Invasive Flowering Shrub on the Pollinator Community of Native Forbs
Source: PLoS One. 2014 Oct 24;9(10):e109088. doi: 10.1371/journal.pone.0109088 (PMC4208741; doi:10.1371/journal.pone.0109088)

Supplemental methods: Exotic plant survey

*Exotic plant survey methods*

We conducted field surveys of exotic plant species in old fields, woodland edges, and prairie remnants near Carlinville, Illinois, USA. The habitats covered in this study are characteristic of habitats experiencing frequent exotic species invasion ([Binggeli 1996](#_ENREF_9)), and provide results with broad implications to similar areas. Between May 9 and July 27, 2009, nine exotic species were identified as focal species for the survey. These exotic species spanned a range of plant families, floral morphologies, mating systems, times since introduction, and life histories (Table S1). We observed 1-3 populations of each species in Carlinville, IL. Each population contained a minimum of ten flowering individuals grown naturally intermixed but dominant compared with co-occurring native and exotic plant species. In each population, we collected insect visitors to the reproductive parts of flowers of the focal exotic species. For this study, we focused on bee visitors (*Hymenoptera, Apoidea*), the most important group of pollinators ([McGregor 1976](#_ENREF_30), [Herrera 1987](#_ENREF_21), [Batra 1995](#_ENREF_8)) and identified them to species. We compared bee visitation rates to each plant species by calculating the catch rate (number of individuals per hour). To compare bee visitor richness among plant species, we performed individual-based rarefaction to control for the different observation times and abundances of pollinators captured from each plant species. Rarefaction sampling was performed using ECOSIM ([Gotelli and Entsminger 2004](#_ENREF_19)).

*Exotic plant survey results*

In total, 439 bees were collected over 95 hours of observation. The richness and catch rates of bee visitors to the nine exotic plants varied greatly (Fig. A1). Raw visitor richness ranged from 3 (*Lonicera maackii*), to 21 bee species (*Trifolium repens*). *Rosa multiflora* had the highest rarefied visitor richness of all exotic species surveyed (15 species, Fig. A1A).  *Rosa multiflora* also received an intermediate catch rate compared to other exotics (Fig. A1B). *Trifolium repens* also received a high diversity of pollinators as well as a high catch rate (Fig. A1). However, *T. repens* thrives in open and highly disturbed habitats where native plant species occur less frequently, and therefore, its effects on native plant-pollinator interactions may be relatively limited. In contrast, *R. multiflora* occurs in forests and forest edges where it co-flowers with native forest understory plants, making it a better candidate for the subsequent removal study.

Supplemental table and figure legends

**Table A1** Exotic plant species surveyed in 2009 and their attributes

**Fig. A1 A)** Rarefied pollinator richness of exotic plants surveyed in 2009 (±SD). *Rosa multiflora* was visited by the highest richness of pollinators. Rarefied means are grouped by 95% confidence intervals, represented by the letters above each bar. The standardized number of bee individuals observed to visit *R. multiflora* was lowest among all plant species observed and used as the standard for rarefaction, thus it does not have an error estimate. **B)** Pollinator catch rates on exotic plants surveyed in 2009. Calculations for each species from data pooled across observed populations.

Table S1

| **Species** | **Family** | **Life history and**  **growth form^1^** | **Presence in the 1880s^2^** | **Floral morphology** | **Breeding system** | **Rounded I-Rank^3^** |
| --- | --- | --- | --- | --- | --- | --- |
| ***Carduus nutans*** | Asteraceae | Biennial/perennial Forb/herb | absent | open | autogamous | High/Low |
| ***Daucus carota*** | Apiaceae | Biennial  Forb/herb | absent | open | animal-pollinated | Low |
| ***Lonicera maackii*** | Caprifoliaceae | Perennial  Shrub | absent | closed | animal-pollinated | High |
| ***Melilotus alba*** | Fabaceae | Annual/biennial/perennial  Forb/herb | present | closed | animal-pollinated | NA |
| ***Melilotus officinale*** | Fabaceae | Annual/biennial/perennial  Forb/herb | absent | closed | animal-pollinated | Med |
| ***Rosa multiflora*** | Rosaceae | Perennial  Vine/subshrub | absent | open | animal-pollinated | Med |
| ***Securigera varia*** | Fabaceae | Perennial  Forb/herb/vine | absent | closed | animal-pollinated | High |
| ***Trifolium pratense*** | Fabaceae | Biennial/perennial  Forb/herb | present | closed | animal-pollinated | Low |
| ***Trifolium repens*** | Fabaceae | Biennial/perennial  Forb/herb | present | closed | animal-pollinated | Med |

^1^USDA PLANTS database (USDA NRCS 2012)

^2^According to presence in the historic dataset of Charles Robertson

^3^U.S. Invasive Species Impact Rank (natureserve.org)

Fig. S1


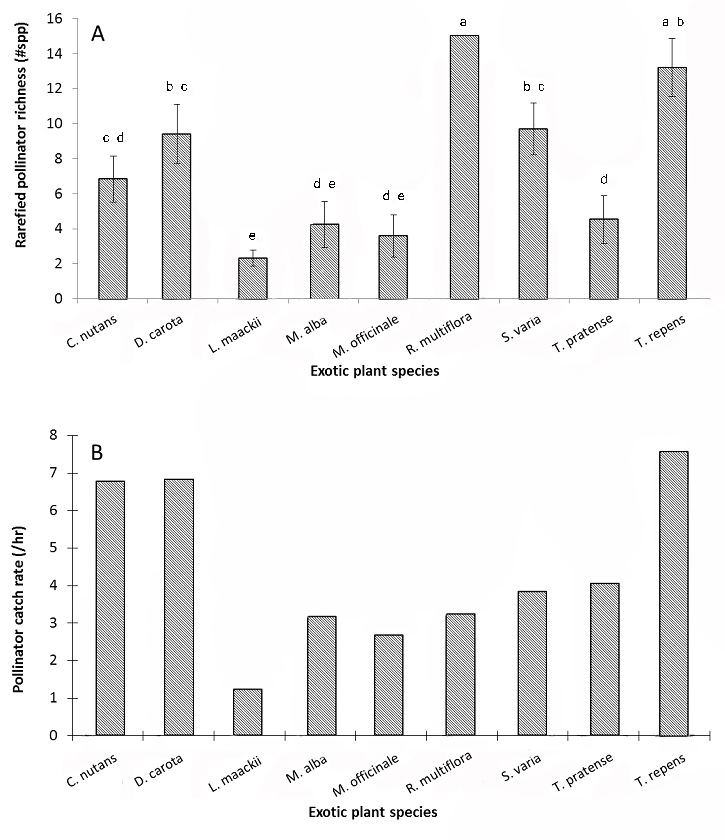

Supplement: Methods S1 — Exotic plant survey methods and results. (DOCX) [file pone.0109088.s001.docx]
